# Information thinking: the transformation of complexity and scientific thinking

**DOI:** 10.3389/fpsyg.2025.1687884

**Published:** 2025-09-17

**Authors:** Tianqi Wu, Haisha Zhang, Kun Wu

**Affiliations:** ^1^School of Humanities and Social Science of Xi'an Jiaotong University, Xi'an, Shaanxi, China; ^2^International Center of Philosophy of Information of Xi'an Jiaotong University, Xi'an, Shaanxi, China

**Keywords:** the philosophy of information, informational thinking, complexity thinking, energy thinking, bifurcation and chaos thinking, teleological thinking, substance thinking

## Abstract

Diverse interpretations of the fundamental nature of the world and its evolutionary patterns have shaped multiple paradigms of scientific and philosophical thinking. At the micro level, ontological understandings have evolved from the ancient Greek atomic theory to modern conceptions based on dynamic energy fields. At the macro level, the comprehension of evolutionary mechanisms has shifted from a teleological model of cosmic evolution to one characterized by bifurcation and chaos in complex systems. The epistemological framework that holds the world to be composed of immutable, indivisible particles with static mass is referred to as “substance thinking,” whereas the view that the world consists of variable, massless energy fields is termed “energy thinking.” While teleological thinking emphasizes stability and simplicity in macro-level evolutionary outcomes, bifurcation and chaos thinking highlights the variability and complexity inherent in such processes. Across both foundational constituents and evolutionary models, scientific cognition has demonstrated a clear transition from simplicity to complexity, thereby driving a paradigm shift in scientific thinking from simplicity-oriented to complexity-oriented approaches. However, the intrinsic multidimensionality and non-linearity of complexity render it incommensurable with any single or limited set of dimensional metrics. The dialectical integration of micro-level randomness, variability, and interaction with macro-level emergence and constructive dynamics may represent the core feature of complexity. On this basis, informational thinking—grounded in the theoretical framework of information philosophy—emerges as a cognitive paradigm that interprets the essence of phenomena through structural, relational, and processual dimensions. By means of symbolic representation, it reveals the historical states, operative mechanisms, and prospective trajectories of systems, thereby offering a novel theoretical dimension—both holographic and integrative—for the advancement of complexity studies.

## 1 Introduction

The worldwide wave of scientific revolution rising in the 20th century shows the general trend of human science from the era of analysis to the era of integrated development, which has taken on a new trend of convergence at a higher level of integration between the end of the 20th century and the beginning of the 21st century. It is the rise of complex information system theory with complexity as the main research object.

There is no doubt that it is necessary for a new research perspective and a new way of thinking to study complexity problems. Although the development of science in the 20th century started the new way for complexity research, it is impossible for the perspective and mode of thinking embodied in any single research program (such as system science research program, information science research program, self-organizing science research program, etc. (Wu, 2010)). Among the many comprehensive research trends rising in the 20th century to undertake the complexity subject of the study of complexity issues. Complex problems indicate that a new research program must have a more complex, comprehensive, profound, and unique nature that transcends individual research programmes that existed in the 20th century.

This new research program is the research program of complex information system that is based on the organic unity of the above scientific research programs, especially the information thinking mode contained in it.

Research on informational thinking in Chinese academia initially emerged from investigations into the relationship between information and thinking within the framework of information philosophy. As (Wu 1985) proposed, “The essence of thinking lies in the subjective creation of information; it constitutes a higher form of material informational activity based on the processing and transformation of information-in-itself.”

In July 1995, the National Symposium on Information Science, Technology, and Philosophy was held in Beijing, China. At this conference, Wu presented a seminal paper titled The Informatization of Science (Wu, 1997a). In his keynote address and accompanying paper, Wu emphasized the distinctive nature, significance, and epistemological value of contemporary information science as a new scientific paradigm.

The paper argues that information science has evolved beyond being a single, independent discipline or merely an interdisciplinary or cross-disciplinary field. Instead, it has developed into a multilayered and comprehensive disciplinary system that spans numerous academic domains. Its most general and universal theories and methodologies represent a new scientific paradigm—one marked by strong penetrability, integrative capacity, and transformative power. When these principles and methods are extended and applied to existing traditional disciplines, they can immediately initiate a comprehensive reconfiguration of those disciplines, endowing them with new epistemological significance. To date, no traditional academic field has proven entirely immune to the influence of the core concepts and general principles of information science.

Wu further points out that science, like society, the economy, and everyday life, is undergoing a process of comprehensive informatization in the information age. This transformation of scientific development may be more precisely described as the informatization of science. The development of information science provides a holographic and systemic lens through which the world and its various domains can be understood. Through this lens, information science offers interpretations of the world grounded in its own intrinsic properties and normative frameworks. From the perspective of its overarching theoretical structure, information science constitutes a paradigm shift—one that leads to the construction of a modern scientific system in which information theory serves as the dominant mode of cognition.

In 1997, Chinese scholar (Yang 1997) formally introduced and began to elaborate on the concept of information thinking. He argued that the discovery of the “information world” had fundamentally reshaped human epistemology, replacing the traditional dichotomy of matter and energy with a trichotomous framework: matter, energy, and information. Yang positioned information thinking as a modern cognitive paradigm and highlighted its affinity with traditional Chinese intellectual traditions. He stated that “the distinctive characteristic of Chinese thinking, which sets it apart from other cultural paradigms, lies in its greater emphasis on information thinking.”

(Wu 1999) positively evaluated Yang's perspective, affirming that many classical doctrines within Chinese philosophy—such as the Five Elements (Wuxing), Yin-Yang, Bagua, the dialectic of Being and Non-being (You-Wu), Meridian theory, Zang-Fu theory, correspondence between Heaven and Humanity (Tianren Xiangying), and the unity of Heaven and Humanity (Tianren Heyi)—all reflect a holistic and integrative understanding of the world. Wu argued that these traditional frameworks align with the foundational logic of contemporary systems science, and that key philosophical concepts such as Dao (the Way), Qi (vital energy), Shen (spirit), Xiang (image), and Xing (form) can be more coherently interpreted through the lens of information thinking.

Building on these insights, Wu subsequently laid the theoretical foundation for information thinking through a trilogy of seminal papers (Wu, 2002a,b, 2003). In these works, he articulated a three-stage transformation of human scientific cognition: from material thinking, to energy thinking, and finally to information thinking. He argued that this evolution marks three major epistemological leaps in the development of scientific thought.

In these three foundational papers, Wu clearly defines information thinking as follows (Wu, 2002a):

The concept of information represents a general understanding, definition, and cognition of information as a fundamental form of existence distinct from matter and energy, encompassing its essence, mode of existence, significance, and value. Based on this understanding, information thinking is a cognitive approach that grasps and describes the essence, characteristics, and attributes of existing entities by analyzing their structural organization, relational networks, interactive patterns, and evolutionary processes. This approach regards the structures, relationships, and processes of entities as carriers or codes of information. It aims to decode the implicit content embedded within these carriers—such as an entity's historical states, current mechanisms, and future trends. Additionally, information thinking involves the symbolic re-encoding of real-world objects or information entities through artificial symbolic systems, assigning them specific formal relations. This comprehensive cognitive method constitutes the mode of informational cognition, known as information thinking.

Information thinking, as a concept of complexity, constitutes a novel and integrative scientific cognitive approach distinct from micro-level immutable substance thinking and macro-level invariant teleological thinking. It offers a multidimensional, compatible, and holographic-integrative framework that advances the study of complex phenomena.

## 2 Substance thinking: the concept of simplicity based on micro-invariant mass particles in world construction

Whether the nature of the world is simple or complex has always been a controversial issue among philosophers and scientists of all ages.

The oldest simplicity concept for humans about the nature of the world was born in the simple element theory of ancient natural philosophy. The concrete embodiment of this ancient concept of simplicity is to recognize and strive to pursue some or some of the most basic entity elements that make up the world with rigid invariant traits. It gives us grounds to call this cognitive concept the simplicity concept of micro-invariance, that is simplicity thinking.

The atomic theory of ancient Greek philosophers Democritus and Epicurus was the highest achievement of the simple entity elements theory in ancient Greece that provides a basic framework for the development of human substance thinking, that is, the simplicity concept of micro-invariance: the countless atoms moving in the empty space are the origin of the world; Atoms can not be divided, no longer change, birth and death, and with basic properties of size, shape, and weight. The gist of the theory of entity realism, which is based on substance thinking is that the world is made up of various kinds of changeless particles with weight.

That ancient philosophers revealed the simplicity of the nature of the world with a variety of rigid and immutable substance elements does not mean that they did not recognize the existence of complex things in the world. They just insisted that the complex things are aggregated by a variety of simple elements, the differences in the types and proportions of elements and the differences in the way they are combined are the roots of the diversity of things. The simplicity concept of micros-invariance established based on this substance thinking implies such a creed: the micro things are constant and simple while the macro things are variable and complex. As some scholars have pointed out (Görnitz, 2016), for a long time, relevant researchers have always aspired to restore complicated processes and complicated structures to simple processes and simple structures. For thousands of years, natural philosophy and natural sciences have been dominated by the concept that by dismantling the real objects into ever smaller parts one finally will arrive at something indivisible and simple, therefore elementary. Those ultimate, indivisible, material build blocks (atoms, from the Greek word “atomos”) should be so simple as to be completely comprehensible and allow one to construe from them all the complex objects we encounter in the real world.

It was the oldest simplicity concept for humanity about the nature of the world conceived in ancient human philosophy and based on a variety of rigid and immutable substantive elements that established the initial thinking paradigm for Dalton's “atomism” in modern science and trend of thought of “Reductionism” characterized by Analytical thinking.

The trend of thought of “Reductionism,” which prevailed in modern science, is based on the simplicity thinking of micro-invariance. At that time, scientists were convinced that all things and phenomena in the world could eventually be explained at the atomic and molecular levels and all the disciplines in science can eventually be unified into the physical science about atoms and molecules. On this occasion, there was the brilliance of Newtonian mechanics based on the movement of rigid invariant particles and the birth of Laplace's demon.

In addition, of particular concern is that the methodological feature of “mathematical reduction” in this “reduction theory” trend in modern science, that is, all natural phenomena can be reduced to mathematical formulas, and can be described and expressed by mathematical principles and laws. Newton's great work Mathematical Principles of Natural Philosophy highlights this methodological feature. The reason why this kind of mathematical method can be widely accepted and used is that it has been deeply influenced by the following fundamental beliefs of scientists and philosophers for a long time, that is, “Nature likes simplicity” (Natura simplicitaten amat); “She likes unity”(Amat illa unitatem); “She will never sit around but make an unnecessary move”(Munquam in ipsa quicquam otiosum aut superfluum exstitit); “Nature always does things in an easier way, and never going to take a detour” (Natura semper quod potest per faciliora, non agit per ambages difficiles; Burtt, 2018). The simplicity of mathematical equations and the harmony of mathematical concepts fully meet the rational spiritual needs of tracing the source and the aesthetic needs of seeking a harmonious order of natural philosophers.

Certainly, this methodology also has its profound historical roots, that is, the ancient Pythagorean School's “The nature of the world is the numbers” and the geometric interpretation of the generation and evolution of the universe fully embodied in Plato's Timaeus. However, at that time, the “number” or “geometry” reflects more ontological connotations and characteristics.

## 3 Energy thinking: the concept of micro-variable complexity dissolving static mass

However, the glorious era of the substance thinking and the simplicity concept of micro-invariance, initiated by the atomic-molecular theory of modern science, did not last long. By the end of the 19th century, due to the discovery of X-rays, radioactivity, electrons, and radium, especially the subsequent relevant research, the inner structure of the atom is stripped layer by layer, which led to the negation of the theory of substantive structure theory represented by modern atomism. Our ancient concept of entities, the concept that particles with mass construct the world, the concept of micro-invariance and the corresponding thinking mode began to waver in the new scientific context. However, the complexity concept of micro-variability based on energy concepts and corresponding energy thinking that eliminated the mass begins to emerge and rise.

At the beginning of the 20th century, the birth of the theory of relativity and Quantum Mechanics and the follow-up development of its related theories brought an end to traditional substance thinking.

The theory of relativity founded by Einstein gave a generalized extension to the electromagnetic field theory of Faraday and Maxwell and proposed a kind of reality–field, which has energy and momentum, but no static mass and is essentially different from the entity. It can not be regarded as the entity that is made up of particles described by Newtonian mechanics that can only move mechanically any longer. And it can no longer be seen as an atom in the sense of Democritus or Dalton or more micro particles with static mass. According to the theory of relativity, the field form of reality is more basic than the entity form of reality since all entity substance with static mass can be transformed into fields without static mass under certain conditions and relations.

The development of quantum mechanics had unraveled the mystery of the micro-world step by step. So far, more than 300 kinds of elementary particles have been found. However, under the influence of the substantive thinking, parts of scientists are still striving to find the “original substance of the universe.” Their profound idea was: all various elementary particles should be attributed to a few “most basic” particles with entity meaning. However, the observations and experiments of quantum mechanics have repeatedly revealed the following: numerous elementary particles cannot be restored to a few “most basic” elementary particles. They can only be reduced to some radiation, light, waves, or strings that do not have static mass, that is, they are not objects, and can only exist and move as energy.

In 1932, Anderson confirmed the existence of the positron predicted by Dirac in the cosmic ray with the photos taken in the cloud chamber. In 1933, Tibod confirmed that positrons and negative electrons could be annihilated with each other, without leaving entities during annihilation but only emitting γ rays. Later, Anderson found that when γ rays suddenly disappeared, a pair of positive and negative electrons appeared. The discovery of positrons indicates for the first time that there are two types of particles in nature, that is, positive particles and antiparticles that can be created at the same time by getting enough energy and can mutually annihilate after releasing energy when encountering. In subsequent studies, scientists have discovered antiproton, antineutron, anti-Sigma negative hyperons, and so on. Now, according to the theory of quantum mechanics, any particle will have antiparticles that annihilate each other. Scientists predict that there may also be antimatter formed by antiparticles and anti-world composed of anti-life.

Quantum mechanics also indicates the existence of a virtual particle that can realize the exchange of practical energy, but unlike real particles, virtual particles can not be detected by particle detectors. Under certain conditions, virtual particles may also be transformed into real particles and detected by the detector. A further theoretical description holds that even the so-called “empty” space is full of infinite energy since it's a world with virtual particles and annihilated particles are hidden in it. And, in a specific scenario, these seemingly “empty” spaces will erupt through some kind of instability mechanism to create a visible world composed of measurable energy and mass.

In the middle of the 1980s, string theory, originated in the late 1960s, was received more active attention in the field of quantum mechanics. In this theory, the basic elements that make up the world are no longer particles that take up only a single point in space but something like an infinite string that only has length but no other linearity. These strings can have terminuses (so-called open strings), or they can join each other to be closed circles (closed strings). In string theory, what was originally thought to be a particle is instead portrayed as a wave that propagates through a string, like a wave on the string of a vibrating kite. Besides, since the superstrings on this ultra-microscopic scale are very fine (they have only length, other dimensions including height and width are shrink to zero), they are unable to stabilize and can only exist in the form of infinite oscillations. There is no doubt that the string theory negates cognitive concept of micro-invariant simplicity described by the category of substance, which is obviously more thorough.

The latest development of quantum mechanics theory not only tends to completely negate the concept that takes entities as the initial component of the universe but may also boil the energy of the universe down to “none” or “zero.” Stephen Hawking (Hawking, 1997), a world-famous cosmologist, once wrote in his masterpiece A Brief History of Time: “in quantum theory, particles can be created from particle-antiparticle pairs in energy but it just raises the question of where energy comes from. The answer is that the total energy of the universe is zero. Matter in the universe is made up of positive energy. However, all matter is attracted to each other by gravity. Two pieces of matter that are close to each other have less energy than two pieces of matter that are separated because you have to consume energy to overcome the gravitation that pulls them together to separate them. In this way, in a certain sense, the gravitational field has negative energy. In the situation of a universe that is generally consistent in space, people can prove that this negative energy just counteracts the positive energy represented by matter so that the total energy of the universe is zero.” If Hawking is right, according to the principles of the general relativity universe and the ratiocination made by the Big Bang theory, all the cosmic energy was gathered on an infinite spatial scale at the state of the “singularity” of the universe before the Big Bang. The energy of the universe happened to be zero at that time. This means that the universe where we live was created by “nothing.” Such an extreme deduction disappears the entity as a mark of micro-invariant simplicity (in the annihilation of positive and negative particles, it is converted into energy) and it makes the energy disappear (it reduced to “zero” or “nothing” in the annihilation of positive and negative energy). Some of these new ideas not only completely negate the thinking mode of classical philosophy and classical science but also completely negate the energy concept and energy thinking in classical philosophy and classical science. Maybe we have a last glimmer of hope: a statement of invalidity of general relativity or quantum gravity theory: it loses efficacy at the singularity. Perhaps there will be a new miracle at the singularity that clearly can not be described by our existing science. Of course, we have every reason to ask the following questions: what kind of state in which the energy is “zero” is? What kind of existence will it be transformed into after the annihilation of positive and negative energy?

The contemporary development of quantum mechanics reveals a great difference in the way the microcosm and the macrocosm operate. There are three most important principles: superposition, complementarity, and quantum entanglement. The theory of superposition emphasizes that the motion of micro-particles cannot be described by a unique state. Its operation mode is the superposition of multiple states. When we measure it, we may make one of the states appear. But this kind of presentation has probability and uncertainty. Because of this, the description of the behavior of micro-particles can not simply and directly apply the method of describing macro-objects, and can not be described by the usual deterministic methods such as size, shape, position, trajectory, etc. At the micro level, particles have become pervasive in the vast space as waves. According to this, quantum mechanics emphasizes that micro particles behave both as granular separated particles and cloud-like diffuse waves. It is based on this understanding that people who study quantum mechanics have proposed the so-called “Wave particle duality,” “Uncertainty Principle” and “Complementary principle.” The discovery of quantum entanglement has even puzzled people with conventional thinking. Quantum entanglement shows that particles in entangled states cannot be independent of each other even if they are far apart, and they still have interactive changes that affect each other.

Although the reality or epistemological significance of principles of existence mode and motion state of the micro-world emphasized by quantum mechanics is still controversial in many aspects, however, in any case, in the face of the discovery of radioactive phenomena and a series of achievements of field theory, relativity, and quantum mechanics, those who still adhere to simplicity concept of the invariance of the micro-world are getting harder to make out a good case for themselves. Human exploration of the variability and complexity of the micro-world has also shown infinite charm to scientists. Not only the big world is complex, but also the small world is also complex.

Professor Thomas (Görnitz 2016), who once worked in the institute of physics, The University of Frankfurt, said: “The aim of science is the explanation of complicated systems by reducing it to simple subsystems. According to a millennia-old imagination, this will be attained by dividing matter into smaller and smaller pieces. The popular superstition that smallness implies simplicity seems to be ineradicable. However, since the beginning of quantum theory, it would be possible to realize that the circumstances in nature are exactly the other way round.” The real situation may be that the deeper people's understanding of the world, the more detailed the mechanism of reality that science and technology can touch, the more complex the structure of reality may be, and the more complex the theory or tool for describing reality will be.

## 4 Teleological thinking: the concept of simplicity of the invariance of macroscopic evolutionary outcomes

The cognitive concept of micro-invariant simplicity is consistent with the cognitive concept of macro-invariant simplicity that in science is embodied in the theory of macro- invariant evolution outcomes. In the cognitive model, the ultimate thinking paradigm about the outcomes of system evolution is exactly the basis of the simplicity concept of macro-invariance.

It is worth mentioning that the final results of the dynamic process emphasized by Bertalanffy (Founder of general system theory) are also obviously characterized by the macro-invariant simplicity. (Von Bertalanffy 1987) had focused on explaining three ways of dynamic process evolution: ultimacy—the system develops to a specific final state according to its specific properties and initial conditions. Equal ultimacy—Starting from different initial conditions, the system reaches the same final state in different ways. True ultimacy (purposiveness or Finality)—The current behavior is prescribed by the intended purpose. In Bertalanffy's opinion, the difference between ultimacy and equal ultimacy reflects the difference of evolution mode between inanimate system and living system. The true ultimate system is the characteristic of human behavior.

The model of the future evolution of the universe inferred by modern cosmology based on the second law of thermodynamics and the big bang hypothesis of the formation of the universe also has the characteristics of ultimate thinking.

In 1850, the Second Law of Thermodynamics put forward by Klaus (later restated as the “principle of entropy increase”) and the “Heat Death-Theory” that derived from it give the first ultimate model of macro-invariant evolution outcomes of the system and even universe. He has a classic description of it: “The more the universe approaches this limiting condition in which the entropy is a maximum, the more do the occasions of further changes diminish, and supposing this condition to be at last completely obtained, no further change could evermore take place, and the universe would be in a state of unchanging death (Wu et al., 2020).”

Many hypotheses hold that the universe where we live has oscillated many times between status of extremely high density and extremely thin density. The universe is likely to show its evolution in an infinite cycle of expansion → contraction → re-expansion → re-contraction → .......For example, (Zöllner 1872) believes that the universe is periodic in the sense that all physical processes occur periodically, rather than in the sense that the size of the universe changes periodically with time. He believes that a limited number of substances will not reach infinity in the process of separation at a limited speed, and they must gather again after a limited time interval. This process is similar to a pendulum, which converts kinetic energy into potential energy at approach and potential energy into kinetic energy at separation. The end of the world is also the beginning of the world. In 1922, Soviet cosmologist (Friedmann 1922) analyzed various solutions of Einstein's field equation from a mathematical point of view and pointed out the possibility of a closed circular universe. He believes that the universe is a periodic world, and its radius of curvature varies between 0 and X0. When the radius of curvature changes periodically, the universe becomes a state of eternal oscillation, that is, undergoing a cyclical process of contraction-expansion-contraction. (Tolman Richard 1934), a famous American physical chemist, and mathematical physicist, believes that the explanation of the continuous expansion and contraction of the universe is reasonable. The most appropriate explanation for the time span of cosmic phenomena is to extend from the negative infinity of the past to the positive infinity of the future. At this time, the universe has an infinite past and future.

Compared with the assumption that “our universe comes from a singularity” inevitably exists in the big bang theory, the traditional classical rebound cosmology holds that the universe comes from a space-time of a very small scale, and its formation process is through a big rebound, that is, the pre-existing universe is highly compressed into a space-time of a very small scale after expanding to the maximum, and then re-expansion into a new universe. Our universe is the result of many generations of rebound and expansion.

(Ashtekar et al. 2006a; 2006b) found that the universe began to contract when it expanded to the extreme. When the energy density of matter was compressed to the Planck scale, it did not enter the singularity of the big bang but experienced a quantum bounce. The classical big bang was replaced by quantum rebound.

(Ijjas and Steinhardt 2019) combined the intervals of ekpyrotic (ultra slow) contract with a (non-singular) classical bounce to form a new cosmic cycle theory. The theory holds that the cycle of the universe from one cycle to another has experienced a non-singular classical rebound stage → accelerated expansion stage → ultra slow thermal contraction stage. But even if it is a different description of the cyclical evolution of the universe, the new theory is still essentially describing an established route and mode of cosmic evolution.

If it's true like this, the universe we live in is unlikely to have any novel innovation and complexity on the whole since this kind of hypothesis holds that the overall evolution model of the universe has the character of cyclic invariant simplicity. It can be seen that whether based on Bertalanffy, the second law of thermodynamics or the description of the future evolution model of the universe in modern cosmology, it presents an ultimate and simple thinking feature at the macro level. The system under this mode of thinking always evolves along a certain direction and path, and finally reaches a final state that does not change, or enters a circular evolution process without any innovation.

## 5 Bifurcation and chaos thinking: the concept of complexity of variable macroscopic evolutionary outcomes

The theory of self-organizing systems developed after the 1970s proposed a bifurcated and chaotic model for the future evolution path of the system, which reveals the variability and complexity of the macro-evolution outcomes of things so as to break the simplicity thinking of macro-invariant evolution outcomes of ultimate thinking scientifically.

The theory of self-organizing systems reveals that the future path of an orderly evolution of non-linear systems has the feature of the uncertainty of random bifurcation. The Dissipative Structure put forward by Prigogine holds that the dynamic equations of a non-linear system can have multiple steady-state solutions. One solution corresponds to the equilibrium state, while the rest of the solution corresponds to the non-equilibrium state. The multiple solutions show the bifurcation model of system evolution. Bifurcation means the diversity and complexity of the direction and mode of system evolution.

The theory of chaos reveals that chaos is a dynamic behavior of deterministic systems. Provided that the deterministic system has a slightly complex non-linear, it will produce inherent randomness within a certain range of control parameters. The motion model of the future evolution of the system caused by this kind of inherent randomness will be irregular, aperiodic, and unusually complex. Even if both the initial conditions and the external control parameters have been determined, the future evolution model of the system is still indeterminate. The production of randomness in the system is caused by the autonomous movement of the elements that make up the system and random interaction between elements produced based on this autonomous movement. In this way, the emergence of internal randomness is rooted in the basis of the random variable complexity of micro-elements.

Actually, the reason for the sensitive dependence of the evolution of the system on the initial value (butterfly effect) described in chaos theory is rooted in the complexity of invariance of micro-elements. Even if the measured initial value is exactly the same, since the elements that make up the system itself have the variability of “free will” (dynamic elements), the system will also produce internal condition difference that is enough to cause sensitivity changes in subsequent evolution so as to introduce the diversity and complexity of the future evolution model. In general, there is a statement describing the butterfly effect, that is, “A miss is as good as a mile.” However, what happens in a real system with internal randomness may be as good as a millionth of a percent, while there will also be a thousand miles of error. Because even if two butterflies are completely in the same position and posture in the initial state but every butterfly has its own “free will.” Therefore, it is not possible to stir up wings in the same direction at the same time with the same force in their following actions. In light of this, the bifurcation and chaos of macro-evolution outcomes, the complexity of macro-variable evolution outcomes, and the complexity of micro-variability are the inner unity in the process of the concrete evolution of things. Besides, the latter is just the deep foundation of the former.

If the chaotic system is divided according to its chaotic degree, there are two forms: one is the general (ordinary) chaotic system with predictability in the future. It is characterized by a low degree of disorder and randomness. Its advantage is that although there are many influencing factors on the system, as long as many influencing factors are taken into account as possible, it will infinitely approach an unambiguous number. In terms of applications, such as weather forecasting, more and more influencing factors can be added to it by establishing a model, so that more and more accurate forecasts of the future form of the weather can be made. But for a need to be more strong random and chaotic characteristics of engineering for the general (ordinary) chaotic systems cannot meet their needs, therefore, many scholars from the chaotic system of the general (ordinary) for further research, the second kind of chaotic systems arises at the historic moment: a hyperchaotic system that cannot predict the characteristic state of the future. In 2008, Chinese scholar (Qi et al. 2008) proposed a new model of hyperchaotic systems, Compared with ordinary hyperchaotic systems, this system has an extremely broad frequency bandwidth of high magnitudes, which proves the disorder and randomness of the system anomaly. Moreover, there are abundant bifurcations in different directions of the system, so there are very complex overall dynamics characteristics. In application, for example, for the market, the act of “forecasting” itself is a systematic influence factor, that is, “forecasting” will affect the outcome of the prediction. However, although a hyperchaotic system can not accurately predict the future, it can make people understand the behavior of the complex system and present a variety of possibilities about the future development of the system.

Based on the characteristics of chaotic systems, (Humi 2019) have proposed a model of the bifurcated evolution of the earth's climate that enables the user to experiment by changing the model parameters and exploring the impact that these changes might have on the climate. (Verma et al. 2019) presented a computational approach using bifurcation theory to understand how the pain sensation threshold varies and how it can be controlled. They explored a bifurcation model which is generated in the mathematical model representing the dynamics of this neuron. Through the analysis of this model, people can better understand the mechanism of pain response and provide strategies to control the pain sensation threshold. (Matouk 2020) uses bifurcation diagrams and other tools to confirm the existence of complex dynamics of chaotic attractors in susceptible-infected models for COVID-19 with multi-drug resistance, which can help to understand the complex behavior of severe infectious diseases including COVID-19. Due to the sensitive initial value and strong randomness, chaos, hyperchaos, and new hyperchaos have been widely used in the field of image encryption, which greatly improves the security in the field of information communication (Ping et al., 2018; Ran et al., 2018; Hui et al., 2021).

It can be seen that in some current related research, bifurcation and chaos are not only the characteristics and behavior modes of the system evolution path, but also have been used as a basic method to study various natural phenomena, social phenomena, human perceptional phenomena, and other complex problems and it has great development potential in application engineering.

## 6 The complexity of complexity concept

The general view always regards integrity, protrusiveness, emergence, irreversibility, non-linear, cross-level, and so on as the characteristics of complexity. And then the specific definition of complexity system is constructed. However, the behavior of systems with such characteristics is not always complex.

Integrity, protrusiveness, emergence, etc. all say that the nature of the whole system cannot be produced by the properties of its constituent elements through a simple superposition. It is created as a property of the whole new system. However, whether the created overall property is complex or simple, is deterministic or non-deterministic, is ultimate or non-ultimate, is immutable or variable, is single-mode or multi-mode: this is not clear.

Irreversibility is about the time characteristics of system evolution. This feature can not indicate the simplicity and complexity of the behavior of the system since we can never say that an evolutionary system with reversibility in time is a simple system and the evolution system with irreversibility in time is a complex system. A system that obeys the evolution of the second law of thermodynamics and moves toward equilibrium is also irreversible in terms of time characteristics. The time symmetric reversible system may also be complex. The team led by academician Jiangfeng Du (Wu et al., 2019) of the University of science and technology of China, for the first time in the world, has observed the parity-time symmetry in the quantum world through quantum control. This parity-time symmetry quantum system has wonderful characteristics and is extremely complex.

Normally, non-linearity is regarded as the fundamental dimension to distinguish simple from complex systems. However, a system in which the non-linearity reaches a limit is just a simple system. The system designed by Harken's Synergetics, which is dominated by only one or a few order parameters, does not have complex behavior but the non-linear degree of the system is very high. A highly centralized social system also has an extremely high degree of non-linearity, but is rigid and inflexible, omitting the free will of its members, which is not characterized by complexity. The limits imposed on the overall system prevent complexity in its microconstituent systems.

As for the number of levels of the system, this cannot be a predictor of complexity or simplicity. A multi-level system that achieves the same degree of unchanged rigidity and whose elements lack the degree of freedom also cannot be complex. For example, a highly centralized social system, or a system with strict mental control. The multivariate network system with parallelism, interaction, non-hierarchy, and non-authoritative control may be complex systems, such as the global information network system because all elements (input or output end) that make up the network have their own free will.

The complexity of complexity concept makes it impossible to be defined more strictly by one or a few specific single dimensions. (Turner and Baker 2019) emphasize that the complexity of social systems, driven by self-organization and emergent behaviors, cannot be adequately represented by a single theory or indicator. Similarly, (Bamgboye and Avellán 2025) highlight that while conceptual maps are useful for visualizing system elements and relationships, they fail to fully account for non-linear interactions and emergent phenomena. (Hébert-Dufresne et al. 2024) point out that system-level patterns often emerge from local interactions, which single-dimensional measures cannot reflect, underscoring the need for multi-scale and interdisciplinary approaches. (Vesterby 2008) further warns that reliance on a single metric risks overlooking feedback loops, non-linearity, and dynamic changes, advocating for multi-dimensional approaches that combine quantitative and qualitative methods. Collectively, these studies illustrate that comprehensive, multi-dimensional, and multi-scale approaches are essential for understanding and evaluating complex systems.

However, it does not mean that complexity cannot be recognized or described. In fact, the concept of complexity is associated with the comprehensive integration effect of a series of features such as micro free degree and macro free degree, micro-variability and macro-variability, internal randomness, uncertainty, bifurcation, multi-possibility, fuzziness, as well as chaos, neither absolute linear relation nor absolute non-linear relation; Of course, perhaps the most important comprehensive integration effect should belong to concrete and dialectical unity of micro random variable interaction and macro random variable emergence constructiveness. However, a series of other characteristics or dimensions are meaningful and valuable for the complex behavior of the system only when they serve this concrete and dialectical unified behavior. Here, the understanding of complexity concept and complexity method should adopt a thinking mode of multi-dimensional compatibility, complementarity (including the complementarity and compatibility of difference, intermediary, opposition, diversity, etc.), and holographic comprehensiveness.

## 7 Information thinking: provide a holographic and comprehensive dimension for complexity

The systematic movement in the 20th century had an obvious defect, that is, insufficient attention had been paid to the information factors that made up the world and the value of information factors to the existence and evolution of the system had not been fully understood and excavated. To truly transcend the scientific vision of the 20th century and to realize new synthesis at a higher level, the study of complexity must be paid full attention to the information factors and give it its due position in the 21st century.

The development of information science and information philosophy in the 20th century has cultivated a kind of information thinking mode which is different from traditional substance thinking and energy thinking. Information thinking grasps and describes the essence, characteristics, and attributes of things from the structure and relational model, evolution procedure, and process mode of existing things, takes the structure, relationship, and process of the existing thing as the carrier or symbol of the information, from which the information thinking deciphers the content, significance, and value of the indirect existence about the historical state, realistic relationship and future trend of objects. Furthermore, information thinking will also re-symbolize the real object and the information by humans, and give it a specific representation relationship. Obviously, relevant theories and viewpoints, ways, and methods provided by information thinking can better reflect the general characteristics of holographic synthesis in complexity research so as to provide a new dimension for complexity. In this new dimension, not only the complex behavior of the system can be explained from the perspective of information activities, but also the information activities themselves are complex (Wu and Da, 2021).

When energy thinking that emphasizes micro-variability dispels the substance thinking which advocates the micro-invariance, the foundation that supports the cosmic building is determined by the information coding architectures that integrated by the reality such as waves and strings that with free variability, and field energy with positive or negative properties. This kind of information coding architecture as a new code element constructs a higher level of information coding architecture step by step, producing elementary particles, atoms, molecules in the usual sense, to the general macro object, celestial body, galaxy, and even life, human and human society. It is precisely because the cosmoscopy and macrocosm that we face is based on real information coding framework with complexity of micro free variability that the world we face, and the different levels and ranges of system behavior in this world, show diversity and complexity.

To see the order of the micro information coding structure of things, deciphering the nature, function, significance, and value of the history, status quo, future of things, and the general information content and expression program for the development and evolution of things specified in it, the properties and functions of the holographic mapping of the system as a whole are created in the overall synthesis of the micro information coding framework of the system, exploring or clarifying the specific mechanisms for sudden changes of the system information coding architecture caused by random or accidental micro or macro information interaction or interference, which is a feasible way and method to study the complexity of objects. This research path and method are fully reflected in the following related researches: understanding the key concept of interdependence in biological, economic and social systems through different types of information sharing modes (Rosas et al., 2016); The quantitative relationship of quantum Kolmogorov complexity is derived from the information disturbance relationship (Miyadera, 2011).

By revisiting the analogy between entropy and information, and applying it to the transition state theory proposed in the framework of chemical kinetics, a new concept of structuring information is obtained and be used to analyze the complex phenomena of the development of living organisms (Trambouze, 2006).

The newly developed multimedia technology, information network technology, nerual network technique, gene spectrum reading, splicing, and deciphering techniques, cloning technology, virtual entity technology, nanotechnology, research on irregular fractal and chaos, research on uncertain or asymmetric information economics, research on Natural disasters and social unrest and computer and artificial intelligence research based on atomic, molecular, or quantum components, etc. are rooted in the above research paths and methods reflecting informational thinking. In the sense of information activities, any complex phenomena of evolution of the self-organizing system, such as non-linearity, internal randomness, internal feedback holographic constructiveness, and so on, can be explained by information characteristics, processes and mechanisms (Wu and Nan, 2019).

## 8 Conclusions of the study

Information paradigms (information thinking) revealed by contemporary information science and the philosophy of information may contribute to the way of complex thinking in many aspects. At the very least, we can list the following points:

1. The theory of dual existence and dual evolution of matter and information provides a new information dimension for the concept of existence and evolution of complexity. This dimension inspires us that the research of all objects and things must be explored from the interactive synthesis of the two dimensions of material and information. Instead of exploring from the perspective of a single material (mass and energy) dimension as emphasized by the traditional scientific and philosophical way of thinking (Wu, 2006a).
2. Information thinking reveals the internal structure of things and the information generated by the structure. This means that the way of existence of anything has the open characteristic of non-isolation. An absolutely isolated thing can't derive an intermediary, that is, it cannot have a relationship with the environment. For that matter, absolutely isolated things are impossible to exist. This reveals the complexity of the way things exist from a specific angle. There is no absolute internal isolation without internal relationship, because without internal relationship, it cannot enter, because it cannot enter and cannot be separated, so it is impossible to derive any intermediary to the outside, and at the same time, external objects cannot enter it. Anything will change when it derives an intermediary or accepts external forces, and this changed structure bear in mind some contents of the relevant process, which is the information generated by the structure.
3. The theory that the interaction of matter must be realized through the intermediary of information denies the existence of absolute opposites. Because everything that is absolutely opposite is in isolation. But there is no interaction when each is isolated. The interaction between opposites must be realized through mutual derivation mediation, so that in any unity containing opposites, it can not be a simple structure consisting of binary opposition. For in the absence of an intermediary, there is no unity of opposites, and when there is an intermediary, the absolute isolation of the opposites is broken, in this way, any structure containing the relation of opposites is opposition with the intermediary, rather than absolute or pure opposition.
4. The Contemporary theory of complex self-organizing systems reveals the inherent unity relationship of time and space, and proposes the complex relationship of spatiotemporal transformation of “spatialization of time” and “temporalization of space” in the evolution of interaction. However, if the information dimension is not involved, and the passage of time, the structure of space and the evolution of things, as well as the history of evolutionary information are not investigated in a unified way, then such a time-space view with internal integration of time and space and complex charm cannot be clearly defined and explained in any case. It is the “preservation” of “history in time” in the way of information condensation in the real existence that transforms time into the structure of space, thus reflecting the time dimension of information transformation in space, which has the evolutionary effect of “spatialization of time” and “spatialization of space.” In fact, all theories about evolution, all kinds of evolutionary theories, are evolutionary information theories about the inherent unity of space-time transformation (Wu, 2005a).
5. The multi-level information intermediary construction and virtual theory of cognitive occurrence explained by the philosophy of information reveals the multi-dimensional comprehensive nature of cognitive occurrence. Thus, breaking the unipolarity and simplicity of the traditional epistemology, or the purely natural theory of cognitive occurrence, or the pure subject construction theory of cognitive occurrence. Through information step by step intermediary selection, matching, construct and virtual activity, we can well integrate the natural dimension, physiological dimension, cognitive structure dimension, tool and means the dimension of cognitive occurrence, as well as the conditional dimension of natural and social history, so as to truly reveal the complex characteristics of human cognitive occurrence process and mechanism (Wu, 2006b).
6. Absolute reductionism and absolute holism are both extreme and mechanized research methods. Only by breaking their mechanized extremes and organically unifying reductionism and holism can we reasonably explain the general nature and characteristics of complex things. The structuration theory of code element and code sequence in information science is specific ways and methods to organically combine reductionism and holism. The science and technology of Nano and virtual reality are models that organically combine reductionism and holism based on the rational application of relevant theories of information science. The basic characteristics of the self-organization behavior of complex systems and the general process and mechanism of their specific activities can be more truly explained and displayed only from the level of information activities (Wu, 2005b).
7. The information science research program may provide some of the most basic theoretical paradigms with core theoretical significance for the research of complex systems. The most general and universal theory and method of information thinking mode and information system science is a new scientific paradigm. Science in the information age is facing a comprehensive information development process, which can be more appropriately called “scientific information scientization” (Wu, 1997b).

Perhaps, it is the theory, viewpoint, thought, and method embodied in informational thinking that show the brilliance of the field of human complexity research in the 21st century. Complexity thinking is also reflected in all aspects of the development of contemporary human society, which is widely used as a cognitive tool for the analysis of various social phenomena.

## Data Availability

The original contributions presented in the study are included in the article/supplementary material, further inquiries can be directed to the corresponding author.
